# The Transcervical Approach for Parapharyngeal Space Pleomorphic Adenomas: Indications and Technique

**DOI:** 10.1371/journal.pone.0090210

**Published:** 2014-02-27

**Authors:** Gilad Horowitz, Oded Ben-Ari, Oshri Wasserzug, Noam Weizman, Moshe Yehuda, Dan M. Fliss

**Affiliations:** Department of Otolaryngology, Head & Neck and Maxillofacial Surgery, Tel Aviv Sourasky Medical Center, Sackler Faculty of Medicine, Tel Aviv University, Tel Aviv, Israel; Boston University, United States of America

## Abstract

**Background:**

Head and Neck Parapharyngeal space tumors are rare. Pleomorphic Adenomas are the most common Parapharyngeal space tumors. The purpose of this study was to define preoperative criteria for enabling full extirpation of parapharyngeal space pleomorphic adenomas via the transcervical approach while minimizing functional and cosmetic morbidity.

**Methods:**

The surgical records and medical charts of 19 females and 10 males with parapharyngeal space pleomorphic adenomas operated between 1993 and 2012 were reviewed.

**Results:**

Fifteen patients were operated by a simple transcervical approach, 13 by a transparotid transcervical approach, and one by a transmandibular transcervical approach. Complications included facial nerve paralysis, infection, hemorrhage and first bite syndrome. There were three recurrences, but neither recurrence nor complications were associated with the type of surgical approach.

**Conclusion:**

A simple transcervical approach is preferred for parapharyngeal space pleomorphic adenomas with narrow attachments to the deep lobe of the parotid gland and for pleomorphic adenomas originating in a minor salivary gland within the parapharyngeal space.

## Introduction

The parapharyngeal space (PPS) is often described as an inverted pyramid-like space whose base is at the sphenoid bone and apex at the greater cornu of the hyoid bone. [1] This complex space is anatomically surrounded by numerous structures [2] and divided by the styloid process into the pre- and post-styloid spaces. [3] The rationale behind this subdivision is that the different structures occupying these subspaces can be the source of various tumors with an anatomic space of origin suggestive of their nature. [4] Tumors of the PPS are rare and account for only 0.5% of all head and neck tumors, [5] with benign lesions comprising approximately 80% of them. [6] Salivary gland neoplasms, especially pleomorphic adenomas (PAs), are the most common lesions in the prestyloid space, whereas paragangliomas and schwannomas are the most common ones in the post-styloid space. [7] Surgery is the mainstay of treatment for symptomatic patients. [8] Several surgical approaches have been described for the management of PPS tumors: the more common among them for the excision of PPS PAs are the transcervical, transoral, transparotid transcervical and transmandibular approaches. [9] The surgical approach of choice is that which will maximize exposure for complete tumor resection while minimizing functional and cosmetic morbidity. The aim of this study is to delineate the preoperative criteria that will enable full extirpation of PPS PAs via a transcervical approach and to present our technique for transcervical excisions of PPS PAs.

## Patients and Methods

### Data Collection

We reviewed the medical records of all patients with PPS tumors referred to the senior author (D.M.F) between 1993 and 2012. Follow-up data were obtained for all patients from their clinical record notes, physical examinations and imaging studies. This study was approved by the institutional review board (IRB). Since only medical files were obtained, the IRB has approved this study without the need to obtain a signature on a patient consent form as long as all personal information has remained discrete including facial features and disclosing markings (IRB: TLV-0406-13).

### Patient Characteristics

A computer-assisted search performed by the institutional tumor registry identified 108 patients with PPST. The eligibility criteria for inclusion in this study were confirmation of the diagnosis of PA of the PPS and surgery having been performed as the primary modality of treatment. Excluded were patients with deep lobe parotid tumors with minimal PPS involvement. Twenty-nine patients met the inclusion criteria and their medical charts were retrospectively reviewed to retrieve the data on age at operation, gender, tumor location, history of prior resections, surgical approach, additional treatment modalities received, outcome, and histological confirmation of the diagnosis and surgical pathology.

### Methods

This study was approved by the Tel-Aviv Sourasky institutional review board (IRB: TLV-0406-13). Since only medical files were obtained, the IRB has approved this study without the need to obtain a signature on a patient consent form as long as all personal information has remained discrete including facial features and disclosing markings. Patient records and information have been coded prior to analysis to blind researchers of all personal data.

All cases of PPS tumors are preoperatively evaluated by the surgical and radiological teams in order to assess tumor origin and formulate a strategy for the surgical approach. According to our departmental protocol, patients with pre-styloid tumors suspected as being PPS PAs with a narrow attachment (elaborated in the discussion) of the tumor to the deep lobe of the parotid gland (DLPG) or those appearing with a separating plane of fat between the tumor and the DLPG on imaging studies, meet the criteria for a simple transcervical approach. Patients with broad attachments of the tumor to the DLPG are operated via the transparotid transcervical approach.

The transcervical approach begins with a curvilinear incision within a natural skin crease in the neck, approximately 3 cm underneath the lower border of the mandible, and continues with an elevation of a subplatysmal flap to the height of the mandible. The submandibular gland is gently retracted medially, taking care not to damage the marginal mandibular branch. The sternocleidomastoid muscle is retracted posteriorly, and the accessory nerve and the digastric muscle are identified. The hypoglossal and lingual nerves are identified and preserved, and the facial artery is ligated if this is essential for a safe extirpation of the tumor, although this will not be necessary in most cases. We occasionally transect the tendon of the digastric muscle along with the stylohyoid muscle and the stylomandibular ligament in order to gain wider exposure of the operative field in cases of large or high-positioned tumors. In those cases not indicating transection of the digastric muscle, the approach to the PPST is made superior to the posterior digastric and posterior to the mandible. Extra-capsular dissection (ECD) is used to carefully detach the tumor from the surrounding tissues, bearing in mind that the carotid artery and jugular vein are located nearby. Using a right angle clamp, the tumor together with its stalk is transected from the parotid gland, thus leaving most of the deep lobe of the parotid gland intact. Generous irrigation with hot saline is used for homeostasis and starch cleansing in order to avoid tumor residue.

The transparotid transcervical approach starts with the attachment of the patient to a facial monitor. A standard modified Blair incision is performed. Superficial parotidectomy is carried out next. After full exposure of all facial nerve branches, the deep lobe is dissected. The neck incision can be elongated as necessary in cases that the initial incision does not provide adequate exposure to the PPS. Since this approach endangers the facial nerve and is associated with adverse affects such as Frey's syndrome, we reserve it as mentioned for cases with broad attachments as pre determined by imaging studies.

The transmandibular approach is rarely used, but is rather reserved for tumors necessitating wide exposure of the PPS, such as very large lesions, malignant transformations, revisions and masses that were exposed to radiotherapy. [9]

The transoral approach is reserved for very small tumors since it has some salient disadvantages, one of which is harboring a major risk in cases of major bleeding since control of the external carotid artery traversing the PPS is extremely difficult. Additionally, the fact that the surgical field is narrow can limit the extent of dissection and lead to the possibility of an incomplete tumor resection as well as to spillage. In contrast, the Da Vinci system offers the advantage of wide vision and superior precision that may overcome those drawbacks and enable a safe and efficacious transoral excision. [10]

### Statistical Analysis

Statistical analysis was performed using SAS software version 9.2 (SAS Institute Inc, Cary, North Carolina). Comparison between groups of patients was performed using the Mann-Whitney test for continuous variables and the Fisher's exact test for categorical variables. Time to first recurrence was plotted using Kaplan-Meier charts and compared between groups using the Log-Rank test.

## Results

Twenty-nine patients underwent surgical resection for PPS PA between 1993 and 2012. There were 19 females and 10 males whose mean age at surgery was 46.4±14.6 years (range, 12–67 years) and mean follow-up was 41.7±10.5 months (range, 8–97 months). Three surgical approaches were used. A simple transcervical approach was chosen for 15 cases (52%), a transparotid transcervical approach for 13 cases (45%), and a transmandibular approach in one case (3%).

The surgery-related complications included one permanent marginal mandibular nerve paralysis, one wound infection and one first bite syndrome in the transparotid transcervical group, and one episode of hemorrhage in the transcervical group (Table 1).

**Table 1:** Surgical Complications.

Complication	Transcervical (n = 15)	Transparotid (n = 13)	Transmandibular (n = 1)	*P* Value
Facial Nerve Paralysis (permanent)	0	1 (marginal mendibular N.)	0	NS
Infection	0	1	0	NS
First Bite Syndrome	0	1	0	NS
Hemorrhage	1	0	0	NS
Frey's Syndrome	0	0	0	NS

NS  =  Non Significant, N  =  Nerve.

Twenty-six of the patients with PPS PAs (89.6%) are currently free of disease. There were three recurrences (10.3%), however two patients had recurred after being initially treated in other institutions and therefore they are not considered as being our failures. All three of those patients eventually underwent multiple resections as well as radiotherapy (Table 2): one underwent three consecutive operations due to local dissemination and recurrence and was finally referred for radiotherapy, another had had a transmandibular approach and postoperative radiotherapy for massive recurrence after having been operated in another institution, and the third was referred to us with diffuse local recurrence after having been operated twice and then irradiated in another institution. The first two patients are alive and without evidence of disease, and the third patient eventually died from carcinoma ex-pleomorphic adenoma.

**Table 2:** Recurrences.

	Transcervical (n = 15)	Transparotid (n = 13)	Transmandibular (n = 1)	*P* Value
Prior Surgery	0	1	1	NS
Recurrences	0	2	1	0.054
Post-op XRT	0	1*	1	NS
Follow - up	38.8	44.1	54	NS

NS  =  Non Significant, XRT =  Radiotherapy.

* One patient excluded due to prior XRT treatment.

Comparison between the simple transcervical and transparotid transcervical approach revealed no significant differences in recurrences and complications (*p* = 0.054 for recurrences).

## Discussion

**Figure 1:**
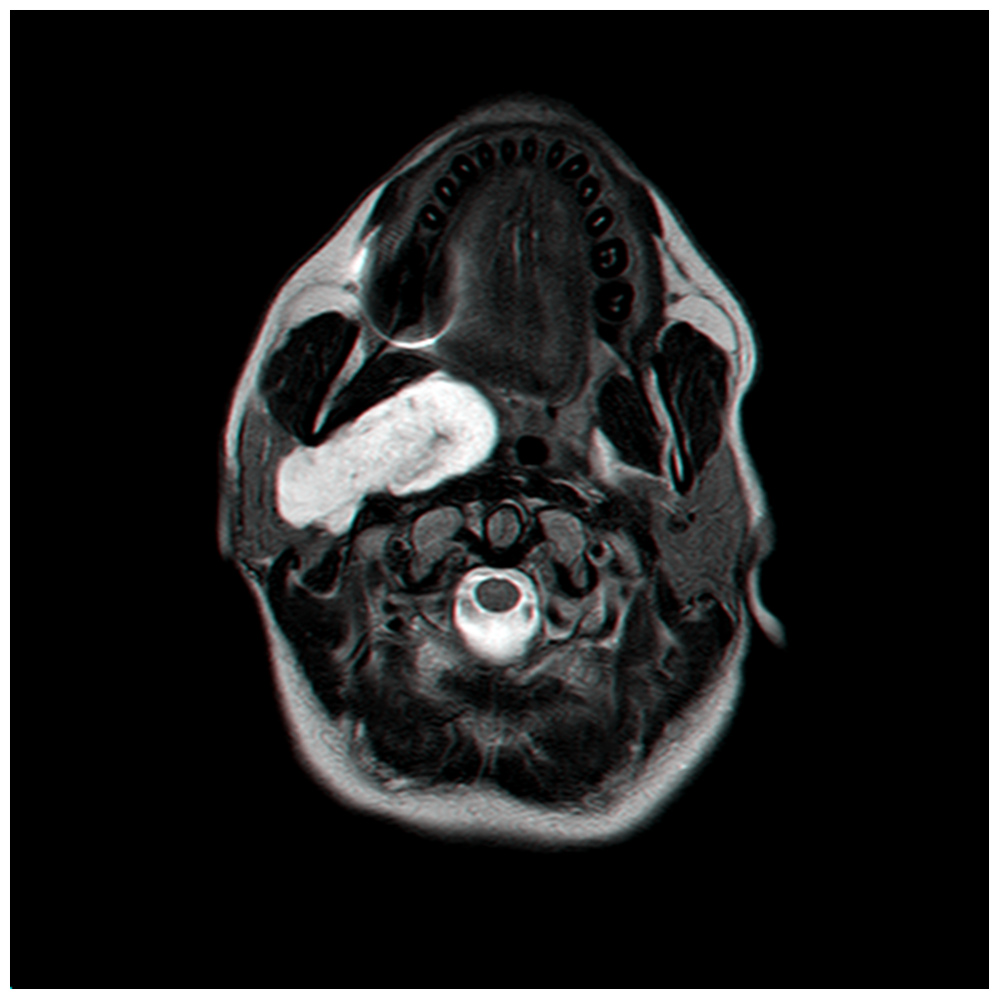
PPS PA with a broad attachment to the DLPG.

**Figure 2:**
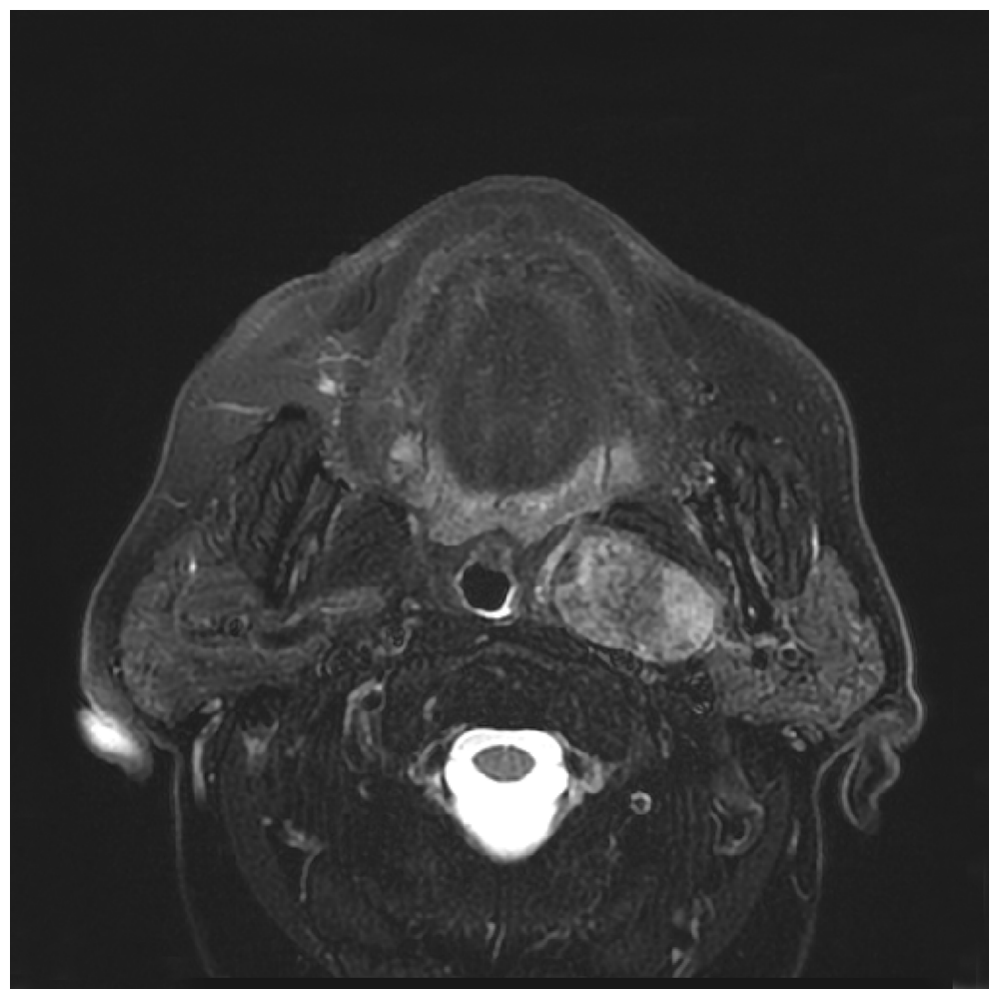
PPS PA with a narrow attachment to the DLPG.

Many authors favor the transparotid transcervical approach for extirpating PPS PAs arising in the DLPG, [6], [11], [12] whereas others advocate the simple transcervical approach because it provides excellent local disease control with minimal risk for facial nerve injury.[13] Preoperative determination of tumor attachment to the DLPG can serve as a crucial criterion for guiding surgical management. Radiological definitions determined by us consists a dichotomy of narrow and broad attachment PPS PAs. All pedunculated appearing tumors not occupying the majority of the DLPG and subjectively seeming to be intra-operatively circumvented with a right angle forceps are defined as narrow attachment tumor whereas all others are considered broad attachment PPS PAs. Tumors that harbor a broad interconnecting margin with the DLPG (figure 1) should be dissected via the transparotid transcervical approach, whereas those with a narrow attachment can be dissected solely through the neck (figure 2). As the dissection approaches the insertion to the DLPG, a right angle clamp is inserted to excise a small cuff of parotid deep lobe parenchyma. This will excise the entire tumor and possible pseudo-pod extensions with: 1) minimal bleeding 2) not endangering the facial nerve 3) shorten operating time and 4) yield better cosmetic results than the transcervical transparotid approach.

By not excising the submandibular gland but rather gently retracting it, the surgeon shall not only preserve the gland but also protect the marginal mandibular branch. In the rare occasion where further exposure of the operating field is necessary, it can be facilitated by transection of the digastric muscle, stylohyoid muscle, stylomandibular ligament and ligation of the facial artery.

There is close agreement among surgeons regarding the full excision of PAs as opposed to simply enucleating them, with mounting new evidence for ECD as being the preferred technique both in superficial parotid gland tumors and PPST.[13], [14], [15], [16] One of the major concerns about the use of ECD is the possibility of tumor spillage. This is traditionally viewed as increasing the risk of tumor relapses in cases of PAs. A historical cohort that addressed this issue was reported by Hughes et al [17] who documented tumor rupture in 10 of the 68 (15%) excised PPS PAs, with recurrence occurring in only one case during a follow-up spanning as long as 27.7 years. Another study on ECD of PPS tumors was recently described by Yang et al. [18] who reported neither recurrences nor complications in 11 patients with PPS PAs excised by means of ECD with a median follow-up of 69.4 months, once again confirming ECD's excellent long-term results. A possible histophathological explanation to the fact that deep lobe originating parotid tumors rarely recur after ECD can be found in an historical cohort comparing capsule thickness among superficial and deep lobe parotid PAs.[19] This cohort found that the capsules were significantly thicker and less likely to be penetrated by tumor in the deep lobe group.

Another important issue concerns PPS PAs not originating from the DLPG but rather minor salivary glands occupying this space. This important distinction could also be recognized preoperatively by high-resolution imaging that reveals a fat plane between the deep lobe of the parotid gland and the tumor. Since even the best modality with the highest possible resolution may miss a small insertion, it is essential to make every effort to determine whether an attachment does exist and possibly excise a small cuff of the DLPG, regardless of negative preoperative imaging findings, in order to circumvent small overlooked attachments during ECDs.

One major drawback aside from known limitations of retrospective analysis is the rather short follow-up time as PA's are notorious for their tendency for long-term recurrences. Nevertheless, it is safe to generalize that as far as PPS PA's, they tend to recur before the five year mark.[15]

## Conclusion

Our results encourage us to prefer the described simple transcervical technique for excising PPS PAs with narrow attachments originating in the deep lobe of the parotid gland.
